# Draft Genome Sequence of *Pedobacter* sp. Strain V48, Isolated from a Coastal Sand Dune in the Netherlands

**DOI:** 10.1128/genomeA.00094-14

**Published:** 2014-02-27

**Authors:** Adam S. Bitzer, Paolina Garbeva, Mark W. Silby

**Affiliations:** a Department of Biology, University of Massachusetts Dartmouth, North Dartmouth, Massachusetts, USA; b Department of Microbial Ecology, Netherlands Institute of Ecology (NIOO-KNAW), Wageningen, The Netherlands

## Abstract

*Pedobacter* sp. strain V48 participates in an interaction with *Pseudomonas fluorescens* which elicits interaction-induced phenotypes. We report the draft genome sequence of *Pedobacter* sp. V48, consisting of 6.46 Mbp. The sequence will contribute to improved understanding of the genus and facilitate genomic analysis of the model interspecies interaction with *P. fluorescens*.

## GENOME ANNOUNCEMENT

*Pedobacter* sp. strain V48 is a Gram-negative, aerobic, rod-shaped bacterium in the family *Sphingobacteriaceae*, isolated from a coastal dune in the Netherlands (1) and categorized in the *Pedobacter* genus by analysis of the 16s rRNA gene sequence (2). The genus *Pedobacter* (phylum *Bacteroidetes*) was proposed by Steyn et al. to encompass phenotypically similar heparinase-positive bacteria from the genus *Sphingobacterium*, which also group closely using a polyphasic approach to classification (3). Based on phenotypic and molecular data, there are now 41 species in the genus (http://www.bacterio.net/pedobacter.html [accessed 1/21/2014]). *Pedobacter* V48 was recently shown to participate in the inhibition of fungal growth during close association with strains of *Pseudomonas fluorescens* (4–6). Thus, the strain is of potential value from the perspective of antifungal compound production. Ongoing studies seek to elucidate how *Pedobacter* V48 either produces antifungal compounds or induces production of novel compounds by *P. fluorescens* and to investigate other interaction-induced traits.

Total DNA was extracted from *Pedobacter* V48 using the Wizard Genomic DNA purification kit (Promega). A library of 300 to 400 bp derived from randomly sheared genomic DNA was constructed, and 50-bp paired-end sequencing (Illumina Genome Analyzer II) was carried out at the Tufts University Genomics core, generating 75,349,698 reads. Reads were initially assembled using Velvet 1.1.6 (7), which produced 174 contigs ranging from 133 to 332,922 bp, with an *N*_50_ of 82,534. To reduce the number of contigs, a second assembly using the forward reads only was carried out with the Geneious 5.6 assembler. The largest 859 Geneious contigs and the Velvet contigs were then assembled using Geneious. The combined assembly yielded 34 contigs (*N*_50_, 403,224), with the largest being 808,927 bp (contig 14) and the smallest 1,225 bp (contig 33). To verify the validity and accuracy of the combination assembly, a BamHI optical map was generated (OpGen), against which the 34 contigs were ordered and mapped using Mapsolver. The resulting order is reflected in the numbering of the contigs from 1 to 26. Contigs which did not unambiguously align were assigned numbers 27 to 34. The total size of all contigs is 6,459,990 bp, leading us to estimate the total genome size to be approximately 6.46 Mbp, with a GC content of approximately 38.9%. The genome is considerably larger than that of published *Pedobacter* sp. sequences (8) and also those of the unpublished *Pedobacter* sp. sequences in GenBank. The percentage of GC is similar to those of other *Pedobacter* genomes in GenBank, which range from 34.4% to 44.9%. Annotation was performed using the NCBI PGAP pipeline, which predicted 5,849 protein-coding sequences and 43 tRNAs. The 5S, 16S, and 23S rRNA genes were detected, but since this is a draft the numbers and locations of multiple copies were not determined. The genome sequence will enable functional genomic and RNAseq-based transcriptomic studies of interactions between *Pedobacter* V48 and *P. fluorescens.*

### Nucleotide sequence accession numbers.

This whole-genome shotgun project has been deposited at DDBJ/EMBL/GenBank under the accession number AWRU00000000. The version described in this paper is version AWRU01000000.
